# Dynamics of Hospitalization and Outcomes of Patients with COVID-19, Influenza, and Respiratory Syncytial Virus (RSV) in a Large Hospital

**DOI:** 10.3390/jcm15218483

**Published:** 2026-10-31

**Authors:** Yasmin Maor, Roni Amir, Guy Rom, Lior Shlomov, Assaf Croll, Orna Schwartz, Debby Ben-David, Iris Zohar

**Affiliations:** 1 Infectious Disease Unit, E. Wolfson Medical Center, Holon 5822012, Israel; roniamir21@gmail.com (R.A.); guyrom06@gmail.com (G.R.); lior.shlomov111@gmail.com (L.S.); irisz@wmc.gov.il (I.Z.); 2 Internal Medicine, Gray School of Medicine, Gray Faculty of Medical and Health Sciences, Tel Aviv University, Tel Aviv 6997801, Israel; ac.croll@gmail.com (A.C.); debbyb@wmc.gov.il (D.B.-D.); 3 Laboratory of Microbiology and Immunology, E. Wolfson Medical Center, Holon 5822012, Israel; ornas@wmc.gov.il; 4 Infection Control Unit, E. Wolfson Medical Center, Holon 5822012, Israel

**Keywords:** COVID-19, influenza, respiratory syncytial virus, hospitalization, Middle East

## Abstract

**Purpose:** Epidemiology data regarding coronavirus disease 2019 (COVID-19), influenza, and respiratory syncytial virus (RSV) in the Middle East is scarce. We describe hospitalization dynamics and outcomes across these viruses in hospitalized adults. **Methods:** A retrospective cohort study conducted in a 700-bed hospital in Israel. All hospitalized adults who tested positive for COVID-19, influenza, or RSV between 1 September 2023 and 31 August 2025 were included. Logistic regression models assessed variables associated with mortality. **Results:** Included were 2201 mono-infected adults (938 COVID-19, 1073 influenza, 190 RSV). COVID-19 demonstrated year-round distribution, whereas influenza and RSV showed seasonality. Invasive ventilation rates were 9.0%, 9.5% and 10.0% in COVID-19, influenza, and RSV patients, respectively. Rates of pneumonia after adjustment were more frequent with influenza. Unadjusted 30- and 90-day mortality were 17.3% and 22.7% for COVID-19, 10.7% and 13.1% for influenza and 18.4% and 21.6% for RSV. In multivariable models, virus type was not significantly associated with 30-day mortality, whereas age, male sex and Charlson comorbidity score were. **Conclusions:** Influenza and RSV exhibit distinct seasonality, whereas COVID-19 persists year-round, with periodic peaks. Mortality was substantial with all three viruses. These findings add to the understanding of respiratory viruses and support vaccination of older and comorbid adults against all three viruses.

## 1. Introduction

Acute respiratory infections represent a leading cause of hospitalization and mortality worldwide, particularly among elderly individuals and those with underlying comorbidities [1,2]. Historically, seasonal influenza has been the dominant viral cause of severe respiratory illness requiring hospital admission. The emergence of severe acute respiratory syndrome coronavirus 2 (SARS-CoV-2) in 2019 and the subsequent coronavirus disease 2019 (COVID-19) pandemic dramatically altered the epidemiology of respiratory viral infections, with non-COVID respiratory viruses nearly disappearing during peak pandemic periods, largely attributable to non-pharmaceutical interventions and behavioral changes [3,4].

As COVID-19 vaccination campaigns progressed and immunity in the population increased—both through vaccination and natural infection—the stringent pandemic control measures were relaxed. This was followed by a notable resurgence of seasonal respiratory pathogens, including influenza and respiratory syncytial virus (RSV) [5,6]. RSV, historically considered primarily a pediatric pathogen, has been increasingly recognized as an important cause of severe respiratory illness in older adults and immunocompromised individuals [7,8].

During the first two years of the COVID-19 pandemic, outcomes of COVID-19 patients compared to historical data regarding influenza patients demonstrated significantly worse outcomes for COVID-19 patients, regarding both hospitalization and mortality [9,10]. Comparative data on the severity and outcomes of hospitalization for COVID-19, influenza, and RSV in real-world settings, particularly in the post-pandemic era when these viruses co-circulate, remains limited. Most existing studies focused on individual pathogens or compared COVID-19 outcomes during peak pandemic waves, limiting their applicability to contemporary clinical settings [11,12]. Data regarding these viruses in the post-pandemic years in the Middle East is scarce.

Understanding how these three viruses differ in their seasonal dynamics, the patient populations they affect, and the clinical outcomes they produce is essential for guiding public health policy, optimizing hospital preparedness, and informing vaccination strategies. This study aimed to describe hospitalization dynamics and compare 30- and 90-day mortality among adults hospitalized with COVID-19, influenza, and RSV at a large hospital in central Israel over a two-year period, following the post-pandemic resurgence of respiratory viruses.

## 2. Methods

### 2.1. Study Design and Setting

This was a retrospective cohort study conducted at E. Wolfson Medical Center, a 700-bed hospital located in central Israel, serving a large urban population. The study period spanned two full respiratory seasons: 1 September 2023, to 31 August 2025. This study is reported in accordance with the Strengthening the Reporting of Observational Studies in Epidemiology (STROBE) guidelines [13] (see Supplementary Table S3).

### 2.2. Study Population

All hospitalized patients aged 18 years or older with a positive polymerase chain reaction (PCR) result for SARS-CoV-2, influenza A, influenza B and/or RSV during the study period were included. Patients with a negative PCR for all three viruses were excluded. There were no exclusion criteria regarding comorbidities, ward of admission, or illness severity.

Records whose positive test fell outside the study period, patients younger than 18 years and patients whose age was not recorded were excluded (Supplementary Figure S1). Patients with a positive PCR for more than one of the target viruses during the same admission were classified as co-infected. Co-infected patients are shown as a separate group in the descriptive tables, and all comparisons between virus types (logistic regression and Kaplan–Meier analyses) were restricted to mono-infected patients, so that the virus groups were mutually exclusive; co-infected patients were analyzed as a separate category, in a sensitivity analysis. Influenza A and influenza B were analyzed together as “influenza”.

### 2.3. PCR Testing

As per institutional protocol, all hospitalized patients presenting with fever and/or respiratory symptoms, severe weakness or syncope, routinely undergo multiplex PCR testing for SARS-CoV-2, influenza (A and B), and RSV. Testing was performed using validated molecular assays in the hospital’s microbiology laboratory.

### 2.4. Data Collection

Patient records were manually reviewed and data extracted by trained personnel. Collected variables included demographic data (age, sex); functional status (debilitation defined as dependency in activities of daily living); prior healthcare exposure (hospitalization in the preceding 90 days); Charlson Comorbidity Index score [14]; immune status (immunocompromised, defined as active malignancy receiving chemotherapy, solid-organ or hematopoietic stem-cell transplantation, or receipt of immunosuppressive therapy); baseline oxygen dependency; length of hospital stay, and the need for invasive ventilation. In addition, in-hospital complications (pneumonia, sepsis, septic shock, acute coronary syndrome/arrhythmia, dialysis requirement, deep vein thrombosis/pulmonary embolism, and stroke) and antimicrobial treatment (receipt of oseltamivir, COVID-19-specific antivirals [nirmatrelvir–ritonavir or remdesivir], and any systemic antibiotic during admission) were recorded.

### 2.5. Outcome Definitions

The primary outcome was 30-day mortality from the date of the first positive PCR test; deaths on the day of the positive test were included. Secondary outcomes were 90-day mortality, need for invasive ventilation, and a composite outcome of 30-day mortality and invasive ventilation.

### 2.6. Definitions

Pneumonia refers to clinically and radiologically diagnosed pneumonia, regardless of etiology as defined and documented by the treating physician. Complications present on admission were not distinguished from those developing during hospitalization. Microbiological confirmation of bacterial co-infection was not systematically available. Sepsis and septic shock were defined by the treating physician. In general, this is in accordance with the Third International Consensus Definitions (Sepsis-3) [15]: sepsis as life-threatening organ dysfunction in response to infection, and septic shock as sepsis requiring vasopressor therapy to maintain arterial pressure ≥65 mmHg with a serum lactate >2 mmol/L, despite adequate fluid resuscitation. Patients with missing data for a complication were excluded from the analysis of that complication. Debilitation was defined as dependency in activities of daily living. Baseline oxygen dependency was defined as the use of home oxygen therapy before admission. Invasive ventilation was defined as endotracheal intubation and mechanical ventilation at any time during the index admission. Prior hospitalization was defined as any hospital admission in the 90 days preceding the index admission. Antibiotic use was defined as receipt of any antibacterial agent recorded during the admission.

### 2.7. Statistical Analysis

Categorical variables are reported as counts and percentages. Continuous variables are reported as means with standard deviations (SDs), or as medians with interquartile ranges (IQRs) for skewed distributions. The three mono-infected groups were compared using the chi-square test for categorical variables and ANOVA or the Kruskal–Wallis test for continuous variables, as appropriate. Missing data were handled by complete-case analysis; the number of patients with missing data for each variable is reported in the table footnotes. Weekly counts of positive cases for each virus were computed and plotted to characterize temporal distribution.

Chi-square tests were used to compare the frequency of in-hospital complications across virus groups. Complications that differed significantly between groups (pneumonia, sepsis, and septic shock) were further examined among mono-infected patients, with the same covariates and reference categories as the mortality models; the number of events is reported in the tables using univariate and multivariable logistic regression (reference group: influenza), adjusted for age, sex, immunocompromised status, and Charlson comorbidity score. Separately, univariate and multivariable logistic regression, adjusted for age, sex, immune status, and Charlson score, were used to assess associations between antiviral (oseltamivir, COVID-19-specific antivirals) or antibiotic treatment and 90-day mortality.

To assess the association between virus type and 30- and 90-day all-cause mortality, we used two complementary approaches. First, time-to-death up to 90 days was analyzed using the Kaplan–Meier method, with cumulative mortality curves generated for each virus group and compared using the log-rank test (overall and pairwise). Patients who did not die within 90 days were censored on day 90.

Second, univariate and multivariable logistic regression models were constructed for 30-day mortality, 90-day mortality and the composite endpoint of 30-day mortality and/or invasive ventilation among mono-infected patients. Virus type was entered as a single categorical variable with influenza as the reference group; to obtain all pairwise virus comparisons, the models were re-fitted with COVID-19 as the reference category. Covariates (age, sex, immunocompromised status and Charlson comorbidity score) were selected a priori as established determinants of mortality in hospitalized adults with respiratory infection that differed between the virus groups, while limiting the number of parameters relative to the number of outcome events (≥10 events per parameter); all were entered simultaneously. Reference categories were female sex and non-immunocompromised status. Linearity of the logit for the continuous predictors (age and Charlson score) was assessed using the Box–Tidwell procedure; no departure from linearity was found (*p* ≥ 0.08), and both were modeled as continuous linear terms. Results are reported as odds ratios (ORs) with 95% confidence intervals (CIs) and *p*-values for all variables; an overall Wald test was used to assess the association of virus type with each outcome. Model calibration was assessed with the Hosmer–Lemeshow test and discrimination with the C-statistic. Sensitivity analyses used alternative adjustment sets: (i) adding debilitation, baseline oxygen dependency and prior hospitalization; (ii) adding study year (2023–2024 vs. 2024–2025) to account for calendar time; and (iii) including co-infected patients as a separate virus category. Age-stratified analyses (<65 years and ≥65 years) were performed to evaluate whether the relationship between virus type and mortality differed by age group. The number of observations and outcome events included in each model is reported in the corresponding table.

Analyses were performed using SPSS Statistics (V31) (IBM Corp., Armonk, NY, USA). Statistical significance was defined as *p* < 0.05 (two-tailed).

During the preparation of this work, the author(s) used CLAUDE Opus (5.5) to edit English, assist in statistical analyses and improve figures. After using this tool, the authors reviewed and edited the content as needed and take full responsibility for the content of the published article.

## 3. Results

### 3.1. Patient Population and Temporal Distribution

**Figure 1:**
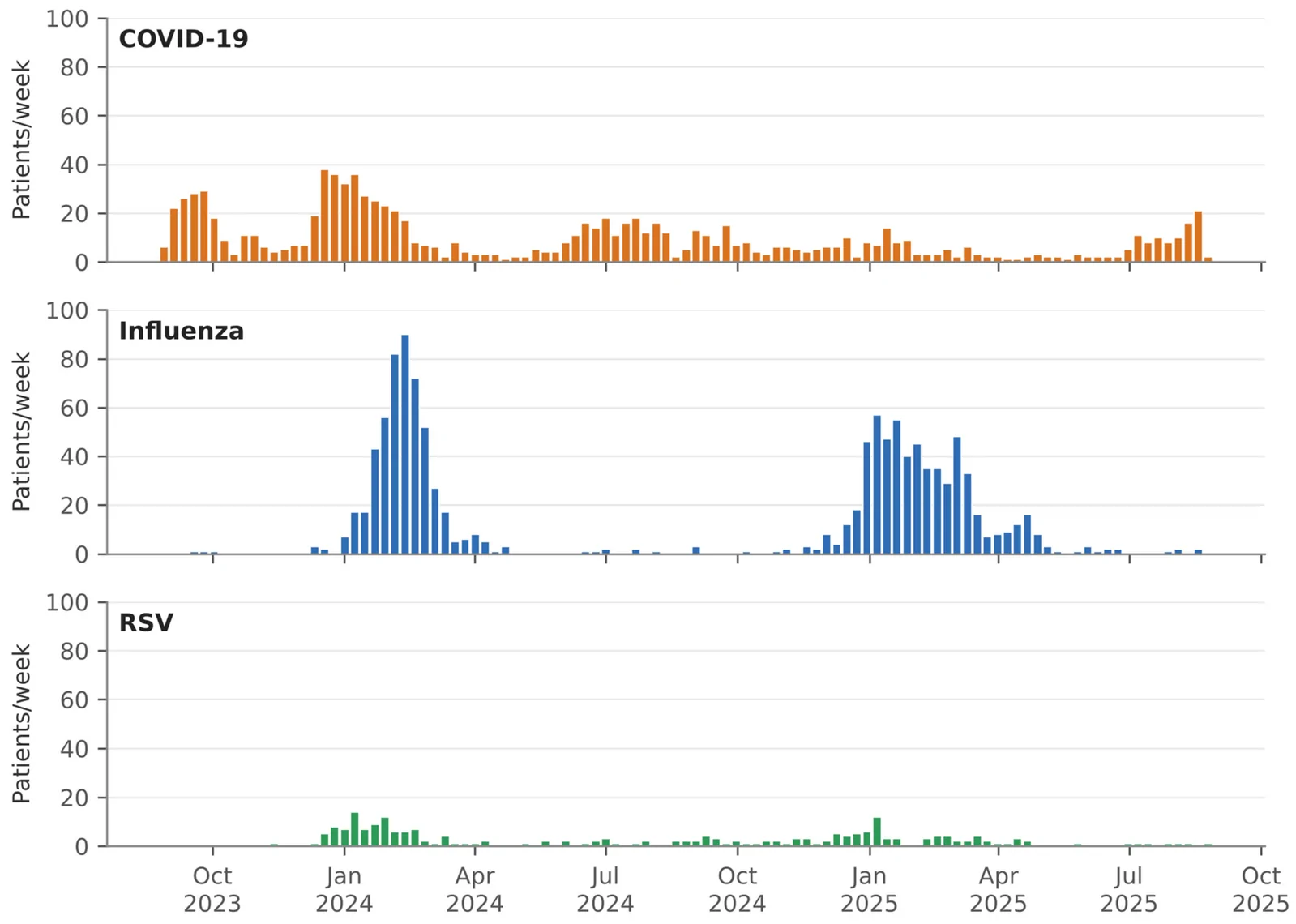
Weekly counts of COVID-19, influenza, and RSV among hospitalized patients at E. Wolfson Medical Center, September 2023–August 2025. Each bar represents the number of adult patients with a new positive PCR per week (co-infected patients are counted in each relevant panel). COVID-19 (orange) shows a year-round pattern with periodic peaks, while influenza (blue) and RSV (green) demonstrate a distinct seasonal winter distribution.

A total of 2276 adults were included (Supplementary Figure S1), of whom 1296 (56.9%) were diagnosed during the first study year (1 September 2023–31 August 2024) and 980 (43.1%) during the second (1 September 2024–31 August 2025). Of these, 2201 (96.7%) were mono-infected: 938 (42.6%) with COVID-19, 1073 (48.8%) with influenza (including 88 with influenza B, who were analyzed together with influenza A) and 190 (8.6%) with RSV. Seventy-five (3.3%) patients were co-infected (49 COVID-19 and influenza, 7 COVID-19 and RSV, 19 influenza and RSV). Figure 1 illustrates the weekly number of patients admitted with each virus over the study period.

COVID-19 cases were distributed throughout the two-year study period with modest peaks occurring approximately every 5–6 months. In contrast, influenza and RSV demonstrated a pronounced seasonal pattern, with most cases clustered in the winter months (November through March), reflecting traditional influenza-season dynamics. The peak influenza season of January–February 2024 was particularly notable, with weekly counts reaching 90 cases.

### 3.2. Patient Characteristics

Characteristics of mono-infected patients by virus type are summarized in Table 1. COVID-19 and RSV patients were older than influenza patients [mean (SD) age 75.0 (16.5) and 75.7 (17.3) years, respectively, vs. 64.5 (21.8) years; *p* < 0.001]. Males comprised 51.6% of COVID-19, 43.6% of influenza and 37.4% of RSV patients (*p* < 0.001).

RSV patients had the highest prevalence of immunocompromised status (36.3%), compared with influenza (14.3%) and COVID-19 (12.8%) patients (*p* < 0.001). Debilitation was also more frequent among RSV and COVID-19 patients than among influenza patients (43.2%, 39.7% and 22.0%, respectively; *p* < 0.001). Charlson comorbidity scores were similar in the COVID-19 and RSV groups [mean (SD) 2.05 (1.69) and 2.07 (1.61)] and lower in the influenza group [1.50 (1.57); *p* < 0.001]. Hospitalization in the preceding 90 days was most common among COVID-19 patients (28.1%), followed by RSV (20.5%) and influenza (17.2%) patients (*p* < 0.001). Rates of invasive ventilation (9.0%, 9.5% and 10.0%; *p* = 0.89) and 180-day re-admission (*p* = 0.31) did not differ significantly between groups.

### 3.3. In-Hospital Complications

In-hospital complication rates by virus type are summarized in Table 2. Three complications differed significantly across virus groups: pneumonia was most frequent in influenza patients (19.6% vs. 15.2% in COVID-19 and 17.4% in RSV, *p* = 0.03), whereas sepsis (7.3% vs. 3.9% and 4.7%, *p* = 0.004) and septic shock (4.6% vs. 2.3% and 2.1%, *p* = 0.01) were most frequent in COVID-19 patients. Rates of acute coronary syndrome/arrhythmia, dialysis, deep vein thrombosis/pulmonary embolism and stroke did not differ significantly between virus groups.

### 3.4. Adjusted Analysis of Complications

After adjustment for age, sex, immunocompromised status and Charlson score among mono-infected patients (*n* = 2169; Table 3), the higher frequency of pneumonia with influenza persisted, relative to COVID-19 (crude OR 0.73, 95% CI 0.58–0.93; aOR 0.57, 95% CI 0.45–0.73) and RSV (crude OR 0.86, 95% CI 0.58–1.29; aOR 0.61, 95% CI 0.40–0.93), indicating that it was not explained by the measured covariates. In contrast, COVID-19’s excess of sepsis (crude OR 1.92, 95% CI 1.29–2.85; aOR 1.51, 95% CI 1.00–2.27, *p* = 0.05) and septic shock (crude OR 2.01, 95% CI 1.22–3.32; aOR 1.59, 95% CI 0.95–2.68, *p* = 0.08), relative to influenza, was attenuated after adjustment, although the point estimates remained above 1. Charlson comorbidity score was independently associated with both sepsis (aOR per point 1.32, 95% CI 1.18–1.47) and septic shock (aOR per point 1.24, 95% CI 1.08–1.42). When debilitation, baseline oxygen dependency and prior hospitalization were added, the pneumonia association was unchanged (COVID-19 vs. influenza aOR 0.53, 95% CI 0.41–0.68), and the estimates for COVID-19 were further attenuated for sepsis (aOR 1.27, 95% CI 0.83–1.93) and septic shock (aOR 1.40, 95% CI 0.83–2.38).

### 3.5. Treatment Uptake

Antiviral and antibiotic treatment by virus type are summarized in Table 4. Antiviral therapy was virus-matched: oseltamivir was administered to 800 (74.7%) influenza patients and COVID-19-specific antivirals to 448 (48.5%) COVID-19 patients, with minimal cross-over; RSV has no approved targeted antiviral, and only three (1.6%) RSV patients received any antiviral. Antibiotic use increased across groups, from 397 (44.8%) COVID-19 patients to 539 (50.3%) influenza and 129 (67.9%) RSV patients (*p* < 0.001), paralleling the older, more immunocompromised profile of the RSV cohort.

### 3.6. Mortality

**Figure 2:**
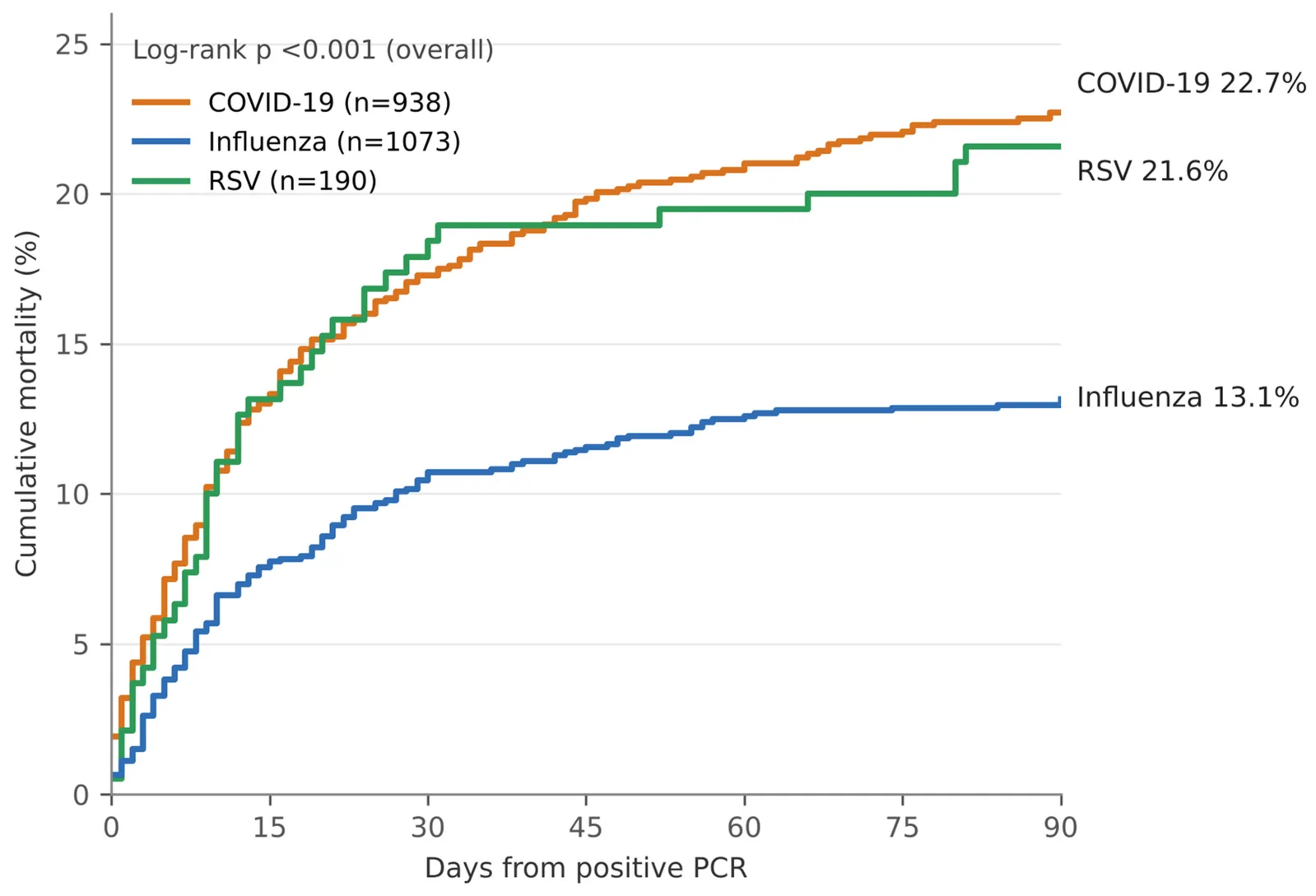
Cumulative mortality up to 90 days by virus type (corrected Kaplan–Meier curves). Cumulative mortality up to 90 days (Kaplan–Meier curves) for mono-infected patients with COVID-19 (orange, *n* = 938), influenza (blue, *n* = 1073) and RSV (green, *n* = 190). Patients who did not die within 90 days were censored at day 90. The COVID-19 and RSV curves largely overlap, reaching 22.7% and 21.6% at day 90, while influenza remains lower, at 13.1%. Log-rank *p* < 0.001 overall; COVID-19 vs. influenza *p* < 0.001, RSV vs. influenza *p* = 0.002, COVID-19 vs. RSV *p* = 0.74.

Unadjusted 30-day mortality was 17.3% for COVID-19, 10.7% for influenza and 18.4% for RSV, and unadjusted 90-day mortality was 22.7%, 13.1% and 21.6%, respectively (Table 1). Figure 2 depicts cumulative mortality over 90 days by virus type (Kaplan–Meier curves). Cumulative mortality differed between groups (log-rank *p* < 0.001): it was higher for COVID-19 (*p* < 0.001) and RSV (*p* = 0.002) than for influenza, whereas the COVID-19 and RSV curves largely overlapped (*p* = 0.74). The composite endpoint of 30-day mortality and/or invasive ventilation occurred in 181 (19.3%) COVID-19, 151 (16.2%) influenza and 41 (25.0%) RSV patients (Table 1).

### 3.7. Predictors of Mortality

Results of the logistic regression models are shown in Table 5, Table 6 and Table 7. In univariate analysis, compared with influenza, 30-day mortality was higher among patients with COVID-19 (OR 1.74, 95% CI 1.34–2.25, *p* < 0.001) and RSV (OR 1.88, 95% CI 1.24–2.85, *p* = 0.003), with no difference between COVID-19 and RSV (OR 0.93, 95% CI 0.62–1.39, *p* = 0.70). After adjustment for age, sex, immunocompromised status and Charlson score, virus type was not significantly associated with 30-day mortality (overall *p* = 0.50; COVID-19 vs. influenza aOR 1.13, 95% CI 0.86–1.49; RSV vs. influenza aOR 1.26, 95% CI 0.81–1.95; COVID-19 vs. RSV aOR 0.90, 95% CI 0.58–1.39). Results were similar for 90-day mortality (overall *p* = 0.16; COVID-19 vs. influenza aOR 1.28, 95% CI 0.99–1.64, *p* = 0.06; RSV vs. influenza aOR 1.17, 95% CI 0.77–1.77) and for the composite endpoint (overall *p* = 0.24). Age (aOR per year 1.05, 95% CI 1.04–1.06), male sex (aOR 1.31, 95% CI 1.01–1.70, *p* = 0.04) and Charlson score (aOR per point 1.26, 95% CI 1.17–1.36) were independently associated with 30-day mortality, whereas immunocompromised status was not (aOR 1.03, 95% CI 0.73–1.44); the same predictors were significant for 90-day mortality. Model calibration and discrimination were adequate (Hosmer–Lemeshow *p* = 0.99 and 0.92; C-statistic 0.74 and 0.74 for the 30- and 90-day models).

In sensitivity analyses (Supplementary Table S1), adding debilitation, baseline oxygen dependency and prior hospitalization did not materially change the estimates for virus type (30-day: COVID-19 aOR 0.99, 95% CI 0.75–1.32, RSV aOR 1.15, 95% CI 0.73–1.80; 90-day: 1.12, 95% CI 0.86–1.45 and 1.07, 95% CI 0.70–1.64), and debilitation was the strongest additional predictor (30-day aOR 3.17, 95% CI 2.38–4.22). Including co-infected patients as a separate category also gave similar results. After additional adjustment for study year, COVID-19 was associated with higher 90-day mortality than influenza (aOR 1.39, 95% CI 1.07–1.81, *p* = 0.01; overall *p* = 0.046), but not with higher 30-day mortality (aOR 1.20, 95% CI 0.90–1.60); 90-day mortality was higher in the second study year (aOR 1.35, 95% CI 1.06–1.73).

In age-stratified analyses (Supplementary Table S2), virus type was not significantly associated with 30-day mortality among patients aged <65 years (*n* = 639; 29 deaths; overall *p* = 0.19) or ≥65 years (*n* = 1531; 278 deaths; overall *p* = 0.86). Among patients <65 years, the point estimate for RSV was high, but very imprecise (aOR 2.92, 95% CI 0.88–9.62).

Associations between treatment and 90-day mortality are summarized in Table 8. Among influenza patients, oseltamivir was associated with higher crude mortality (OR 1.76, 95% CI 1.11–2.80), but not after adjustment (aOR 0.74, 95% CI 0.44–1.24). Among COVID-19 patients, COVID-19-specific antivirals were not associated with mortality (aOR 0.88, 95% CI 0.63–1.22). Antibiotic use remained associated with 90-day mortality after adjustment (aOR 3.38, 95% CI 2.58–4.43) among COVID-19 (aOR 4.03, 95% CI 2.80–5.79) and influenza (aOR 3.33, 95% CI 2.10–5.26) patients, but not among RSV patients (aOR 1.19, 95% CI 0.52–2.72). This pattern is consistent with confounding by indication, with antibiotics marking suspected bacterial infection and illness severity. As these are observational data without treatment-timing or severity-at-initiation information, none of these associations should be interpreted as causal.

## 4. Discussion

In this two-year retrospective cohort study of 2276 adults hospitalized with COVID-19, influenza, or RSV at a large hospital in the center of Israel, we demonstrated three key findings. First, these three viruses had markedly different seasonal dynamics: COVID-19 circulated year-round, with periodic surges approximately every 5–6 months, while influenza and RSV retained a distinct winter seasonal pattern. Second, these viruses infected substantially different patient populations in terms of age, comorbidity burden, and immune status. Third, although crude mortality differed between viruses, these differences were not statistically significant after adjustment, and age, sex and comorbidity burden were the strongest independent predictors of 30- and 90-day mortality and of the composite endpoint of 30-day mortality and/or invasive ventilation.

The persistence of COVID-19 as a year-round pathogen is an important clinical and public health finding. Unlike influenza and RSV, which are constrained by seasonal immunological and environmental factors, SARS-CoV-2 continues to evolve and generate successive waves driven by immune-escape variants, maintaining epidemic pressure throughout the calendar year [16,17]. Data regarding the current seasonality of these viruses in the Middle East during 2024–2025 has not yet been published. Studies from Oman [18], Qatar [19], Jordan [20] and Saudi Arabia [21] relate to data up to 2023 or before. Virus seasonality has significant implications for hospital preparedness, as it means COVID-19 can exacerbate bed pressure at any time, rather than solely during the winter respiratory season, when influenza and RSV simultaneously peak.

The patient populations affected by these three viruses differed substantially. RSV patients were older, on average, and had the highest prevalence of immunocompromised status (36.3%), consistent with growing literature describing RSV as a particularly dangerous pathogen in immunocompromised hosts [7,22]. Influenza patients were younger, on average, and had lower comorbidity scores and the lowest rate of prior hospitalization.

The finding that unadjusted 30-day mortality was highest for RSV (18.4%) and COVID-19 (17.3%), compared to influenza (10.7%), may reflect the older age and higher comorbidity burden in the RSV and COVID-19 cohorts. After adjustment for these factors, the differences in mortality between the three viruses were no longer statistically significant, consistent with several prior comparative studies [23,24]. This suggests that host characteristics, particularly age and comorbidity burden, account for much of the crude difference in mortality between viruses in hospitalized adults. However, this observational analysis cannot fully separate host from pathogen effects: residual confounding by unmeasured factors, such as illness severity at presentation, vaccination status, time from symptom onset and treatment timing, is possible, and the absence of a statistically significant difference does not establish equivalence. After additional adjustment for study year, COVID-19 was associated with higher 90-day (but not 30-day) mortality than influenza, so a modest excess in later mortality with COVID-19 cannot be excluded, perhaps due to declining vaccination. The RSV estimates were imprecise: with 190 mono-infected RSV patients, the study had approximately 80% power to detect a difference in 30-day mortality between RSV and influenza only if it was about 8 percentage points or more (approximately 19% vs. 11%, an OR of about 2), so smaller but clinically important differences cannot be excluded. This study was not designed as an equivalence or non-inferiority analysis, and our findings should not be interpreted as showing that the three viruses carry equivalent risk, or as evidence about the intrinsic virulence of each pathogen. From a practical standpoint, our findings support risk stratification of hospitalized adults with any of these viruses based on age, sex and comorbidity, rather than on the identity of the virus alone.

The age-stratified analyses (Supplementary Table S2) showed no significant association between virus type and mortality in patients aged <65 or ≥65 years, although the smaller strata, particularly the younger RSV group, had limited power to detect moderate differences between viruses.

Beyond mortality, in-hospital complications revealed a similar, though not identical, pattern. Pneumonia was significantly associated with influenza, and this association strengthened after adjustment relative to COVID-19 and RSV, consistent with the well-described phenomenon of bacterial superinfection complicating influenza infection [25]. Sepsis and septic shock showed a pattern similar to that of mortality: COVID-19’s crude excess relative to influenza was attenuated after adjustment, and Charlson score was independently associated with both complications. Taken together, these findings suggest that the excess pneumonia with influenza may reflect pathogen-related factors, such as the well-described propensity for secondary bacterial infection, whereas the crude differences in sepsis and septic shock appear to be explained largely by differences in comorbidity between the groups. Because the adjusted estimates for sepsis and septic shock remained above 1, with confidence intervals compatible with a moderate effect, a virus-specific contribution cannot be excluded.

Treatment patterns tracked the underlying virus, with oseltamivir and COVID-19-specific antivirals showing minimal cross-over between groups. Neither antiviral class was independently associated with 90-day mortality after adjustment: oseltamivir’s apparent crude harm (OR 1.76) is likely explained by confounding by indication, as it was no longer significant once age, sex, immune status, and comorbidity were accounted for. Antibiotic use remained associated with mortality after adjustment, but this most likely reflects the fact that antibiotics were preferentially given to patients with bacterial superinfection or greater illness severity, rather than a causal effect of treatment; the absence of this association among RSV patients, most of whom (67.9%) received antibiotics, is consistent with this interpretation. As these treatment analyses are observational and lack data on treatment timing or severity at initiation, they should be interpreted as hypothesis-generating, rather than causal.

Antibiotics were prescribed to 44.8% of COVID-19, 50.3% of influenza and 67.9% of RSV patients, whereas pneumonia was diagnosed in only 15.2–19.6% and sepsis in 3.9–7.3% of patients. This gap suggests that a substantial proportion of antibiotic courses may have been given empirically, without documented bacterial infection, a pattern described previously in hospitalized patients with COVID-19 and other respiratory viral infections [26,27]. Rapid multiplex PCR, which establishes a viral diagnosis early in the admission, offers an opportunity for antimicrobial stewardship. In patients with a confirmed respiratory virus and no radiological pneumonia or signs of sepsis, avoiding or stopping empirical antibiotics early may be reasonable. Patients with influenza, in whom pneumonia was more frequent in our cohort, may warrant closer evaluation for secondary bacterial infection. The higher antibiotic use among RSV patients, who were older and more often immunocompromised, may reflect both greater clinical concern and the absence of a targeted antiviral. Because our data do not include the indication, timing or duration of antibiotic treatment, or microbiological confirmation of bacterial infection, we could not assess the appropriateness of individual prescriptions.

Our data underscore the importance of preventive strategies targeting all three pathogens. Effective vaccines are available, or recently approved, for COVID-19, influenza, and RSV (the latter primarily targeted at adults ≥60 years and immunocompromised individuals) [28,29,30]. Given the substantial mortality burden across all three viruses, ranging from 13% to 23% at 90 days in this hospitalized cohort, and the strong association of mortality with age and comorbidity, vaccination of at-risk populations is likely to be the most effective preventive measure. National programs recommending annual influenza and COVID-19 vaccination, alongside the newly available RSV vaccines, should be strongly promoted, particularly in older adults and those with significant comorbidities.

This study has several limitations. This is a retrospective single-center design that may limit generalizability, although E. Wolfson Medical Center serves a large and diverse urban population. As an observational study, it is subject to residual confounding. Several important determinants of outcome were not available, including individual vaccination status, illness severity at presentation (e.g., SOFA or NEWS scores and oxygen requirement at admission), time from symptom onset to admission, and the timing of antiviral and antibiotic treatment. Differences between groups in any of these factors could have influenced both the crude and the adjusted comparisons; for the same reasons, the associations between treatment and mortality are subject to confounding by indication, and cannot be interpreted causally. Although functional status and baseline oxygen dependency were available and were included in sensitivity analyses, other aspects of frailty were not captured. Diagnoses of complications were obtained by retrospective chart review, and depend on the accuracy of clinical documentation. The RSV group was relatively small, limiting the statistical power to detect moderate differences between RSV and the other viruses. Co-infected patients (3.3%) were excluded from the comparative regression analyses, although a sensitivity analysis including them yielded similar results. Finally, because vaccination may reduce both the risk and the severity of hospitalization, differences in vaccine uptake between the populations at risk for each virus could have affected our comparisons. National Israeli data for the study period indicate COVID-19 vaccination coverage of approximately 5–10% in the general population and 20–35% in high-risk groups; influenza vaccination coverage of approximately 16–17% in the general population and 50–54% in high-risk groups; and very low RSV vaccination coverage [31,32].

Future studies should prospectively collect vaccination status, severity scores at presentation, and the timing of antiviral and antibiotic treatment, which were not available in this study. Multicenter or national cohorts across the Middle East would provide adequate power for comparisons involving RSV and, if the clinical question is equivalence of risk, should use a prespecified equivalence framework with justified margins. Further research should also evaluate the effect of the newly available RSV vaccines and updated COVID-19 vaccines on hospitalization and mortality in older adults, assess longer-term outcomes such as functional decline and readmission, and test stewardship interventions that use rapid viral diagnosis to reduce unnecessary antibiotic use. Finally, continued surveillance is needed to determine whether COVID-19 settles into a winter seasonal pattern similar to influenza and RSV or continues to circulate year-round.

In conclusion, COVID-19 has become a year-round respiratory virus, while influenza and RSV retain their winter seasonality. Mortality at 30 and 90 days was substantial with all three viruses. After adjustment for age, sex and comorbidity, differences between viruses were not statistically significant, and these host factors were the strongest predictors of death, although residual confounding cannot be excluded. These findings highlight the need for high vaccination coverage against all three pathogens, targeted at older adults and those with significant underlying disease, including in the Middle East region.

## Figures and Tables

**Table 1:** Patient characteristics and outcomes by virus type (mono-infected patients) and among co-infected patients.

	COVID-19 (*n* = 938)	Influenza (*n* = 1073)	RSV (*n* = 190)	*p*-Value	Co-Infected (*n* = 75)
Age, years, mean (SD)	75.0 (16.5)	64.5 (21.8)	75.7 (17.3)	<0.001	64.2 (22.4)
Male sex	484 (51.6)	468 (43.6)	71 (37.4)	<0.001	33 (44.0)
Debilitated	372 (39.7)	236 (22.0)	82 (43.2)	<0.001	21 (28.4)
Hospitalization in the past 90 days	263 (28.1)	184 (17.2)	39 (20.5)	<0.001	23 (30.7)
Charlson score, mean (SD)	2.05 (1.69)	1.50 (1.57)	2.07 (1.61)	<0.001	1.44 (1.49)
Immunocompromised	120 (12.8)	153 (14.3)	69 (36.3)	<0.001	11 (14.7)
Home oxygen before admission	57 (6.1)	74 (6.9)	25 (13.2)	0.002	5 (6.7)
Length of stay, days, median (IQR)	5 (2–9)	4 (2–7)	5 (3–9.8)	<0.001	4 (2–6.5)
Invasive ventilation	84/934 (9.0)	88/926 (9.5)	16/160 (10.0)	0.89	6/73 (8.2)
180-day readmission	241/894 (27.0)	294/1060 (27.7)	61/188 (32.4)	0.31	20/75 (26.7)
30-day mortality	162 (17.3)	115 (10.7)	35 (18.4)	<0.001	12 (16.0)
90-day mortality	213 (22.7)	141 (13.1)	41 (21.6)	<0.001	12 (16.0)
Composite endpoint	181/936 (19.3)	151/934 (16.2)	41/164 (25.0)	0.01	13/73 (17.8)

Values are mean (SD), median (IQR) or *n* (%); for variables with substantial missing data, *n*/*N* with available data (%) is shown. *p*-values compare the three mono-infected groups (ANOVA, Kruskal–Wallis or chi-square test). Missing data (COVID-19/influenza/RSV): Charlson score 15/12/2; invasive ventilation 4/147/30; 180-day readmission 44/13/2; other variables ≤3 per group. Composite endpoint—30-day mortality and/or invasive ventilation.

**Table 2:** In-hospital complications by virus type.

	COVID-19 (*n* = 938)	Influenza (*n* = 1073)	RSV (*n* = 190)	*p*-Value	Co-Infected (*n* = 75)
Pneumonia	142 (15.2)	210 (19.6)	33 (17.4)	0.03	7 (9.3)
Sepsis	68 (7.3)	42 (3.9)	9 (4.7)	0.004	4 (5.3)
Septic shock	43 (4.6)	25 (2.3)	4 (2.1)	0.01	5 (6.7)
ACS/arrhythmia	70 (7.5)	59 (5.5)	14 (7.4)	0.18	7 (9.3)
Dialysis	23 (2.5)	17 (1.6)	3 (1.6)	0.34	1 (1.3)
DVT/PE	13 (1.4)	10 (0.9)	1 (0.5)	0.45	0 (0.0)
Stroke (CVA)	6 (0.6)	4 (0.4)	1 (0.5)	0.70	0 (0.0)

Mono-infected patients: COVID-19 *n* = 938, influenza *n* = 1073, RSV *n* = 190; co-infected *n* = 75. Missing data ≤3 per group for each complication. Chi-square test across the three mono-infected groups; for complications with expected counts <5 (dialysis, DVT/PE, stroke) *p*-values are approximate. ACS—acute coronary syndrome; DVT/PE—deep vein thrombosis/pulmonary embolism.

**Table 3:** Adjusted analysis of pneumonia, sepsis, and septic shock.

Complication/Comparison	Crude OR (95% CI)	*p*	Adjusted OR (95% CI)	*p*
Pneumonia (*n* = 2169; 383 events)				
COVID-19 vs. influenza	0.73 (0.58–0.93)	0.009	0.57 (0.45–0.73)	<0.001
RSV vs. influenza	0.86 (0.58–1.29)	0.47	0.61 (0.40–0.93)	0.02
COVID-19 vs. RSV	0.85 (0.56–1.28)	0.44	0.94 (0.61–1.45)	0.78
Charlson score, per point	–		1.10 (1.02–1.18)	0.01
Sepsis (*n* = 2169; 117 events)				
COVID-19 vs. influenza	1.92 (1.29–2.85)	0.001	1.51 (1.00–2.27)	0.05
RSV vs. influenza	1.22 (0.58–2.55)	0.60	0.92 (0.43–1.96)	0.83
COVID-19 vs. RSV	1.56 (0.77–3.23)	0.21	1.64 (0.79–3.45)	0.19
Charlson score, per point	–		1.32 (1.18–1.47)	<0.001
Septic shock (*n* = 2169; 71 events)				
COVID-19 vs. influenza	2.01 (1.22–3.32)	0.006	1.59 (0.95–2.68)	0.08
RSV vs. influenza	0.90 (0.31–2.62)	0.85	0.73 (0.25–2.15)	0.56
COVID-19 vs. RSV	2.22 (0.79–6.25)	0.13	2.17 (0.76–6.25)	0.14
Charlson score, per point	–		1.24 (1.08–1.42)	0.002

Logistic regression among mono-infected patients, adjusted for age, sex, immunocompromised status and Charlson score. ORs are shown for each pairwise virus comparison (the first-named group compared with the second). OR, odds ratio; CI, confidence interval.

**Table 4:** Antiviral and antibiotic treatment uptake by virus type.

	COVID-19 (*n* = 938)	Influenza (*n* = 1073)	RSV (*n* = 190)	*p*-Value	Co-Infected (*n* = 75)
Oseltamivir	7 (0.8)	800 (74.7)	2 (1.1)	<0.001	21 (28.0)
COVID-19-specific antiviral	448 (48.5)	1 (0.1)	1 (0.5)	<0.001	19 (25.3)
Any antibiotic	397 (44.8)	539 (50.3)	129 (67.9)	<0.001	29 (38.7)

Mono-infected patients by virus type, with co-infected patients shown separately; *n* (%). Any antibiotic: receipt of any antibacterial agent during admission. Missing antibiotic data: COVID-19 51, influenza 2, RSV 0. *p*-values compare the three mono-infected groups (chi-square test).

**Table 5:** Logistic regression of 30-day mortality among mono-infected patients.

Variable	Univariate OR (95% CI)	*p*	Multivariable OR (95% CI)	*p*
Virus type (overall)		<0.001		0.50
Influenza	Reference		Reference	
COVID-19 vs. influenza	1.74 (1.34–2.25)	<0.001	1.13 (0.86–1.49)	0.37
RSV vs. influenza	1.88 (1.24–2.85)	0.003	1.26 (0.81–1.95)	0.31
COVID-19 vs. RSV	0.93 (0.62–1.39)	0.70	0.90 (0.58–1.39)	0.64
Age, per year	1.05 (1.04–1.06)	<0.001	1.05 (1.04–1.06)	<0.001
Male sex	1.45 (1.14–1.85)	0.002	1.31 (1.01–1.70)	0.04
Immunocompromised	1.26 (0.92–1.73)	0.15	1.03 (0.73–1.44)	0.87
Charlson score, per point	1.40 (1.31–1.50)	<0.001	1.26 (1.17–1.36)	<0.001

Univariate and multivariable logistic regression for 30-day mortality. Multivariable model: *n* = 2170, 307 events; all variables shown were entered simultaneously. Reference categories: influenza (virus type), female (sex), not immunocompromised. Overall Wald *p* for virus type = 0.50; C-statistic 0.74; Hosmer–Lemeshow *p* = 0.99. OR, odds ratio; CI, confidence interval. OR—odds ratio; CI—confidence interval.

**Table 6:** Logistic regression of 90-day mortality among mono-infected patients.

Variable	Univariate OR (95% CI)	*p*	Multivariable OR (95% CI)	*p*
Virus type (overall)		<0.001		0.16
Influenza	Reference		Reference	
COVID-19 vs. influenza	1.94 (1.54–2.45)	<0.001	1.28 (0.99–1.64)	0.06
RSV vs. influenza	1.82 (1.23–2.68)	0.003	1.17 (0.77–1.77)	0.46
COVID-19 vs. RSV	1.06 (0.73–1.56)	0.73	1.10 (0.73–1.64)	0.67
Age, per year	1.05 (1.04–1.06)	<0.001	1.05 (1.04–1.06)	<0.001
Male sex	1.46 (1.17–1.81)	<0.001	1.28 (1.01–1.63)	0.04
Immunocompromised	1.44 (1.09–1.91)	0.01	1.20 (0.88–1.62)	0.25
Charlson score, per point	1.45 (1.36–1.55)	<0.001	1.30 (1.21–1.39)	<0.001

Univariate and multivariable logistic regression for 90-day mortality. Multivariable model: *n* = 2170, 388 events; all variables shown were entered simultaneously. Reference categories: influenza (virus type), female (sex), not immunocompromised. Overall Wald *p* for virus type = 0.16; C-statistic 0.74; Hosmer–Lemeshow *p* = 0.92. OR, odds ratio; CI, confidence interval. OR—odds ratio; CI—confidence interval.

**Table 7:** Logistic regression of composite endpoint of 30-day mortality and/or invasive ventilation among mono-infected patients.

Variable	Univariate OR (95% CI)	*p*	Multivariable OR (95% CI)	*p*
Virus type (overall)		0.02		0.24
Influenza	Reference		Reference	
COVID-19 vs. influenza	1.24 (0.98–1.58)	0.07	0.87 (0.68–1.12)	0.29
RSV vs. influenza	1.73 (1.17–2.56)	0.006	1.21 (0.80–1.84)	0.37
COVID-19 vs. RSV	0.72 (0.49–1.06)	0.10	0.72 (0.47–1.09)	0.12
Age, per year	1.04 (1.03–1.05)	<0.001	1.04 (1.03–1.05)	<0.001
Male sex	1.42 (1.13–1.78)	0.002	1.26 (0.99–1.60)	0.06
Immunocompromised	1.41 (1.06–1.88)	0.02	1.11 (0.82–1.52)	0.49
Charlson score, per point	1.39 (1.30–1.49)	<0.001	1.28 (1.19–1.37)	<0.001

Univariate and multivariable logistic regression for the composite endpoint of 30-day mortality and/or invasive ventilation. Multivariable model: *n* = 2008, 368 events; all variables shown were entered simultaneously. Reference categories: influenza (virus type), female (sex), not immunocompromised. Overall Wald *p* for virus type = 0.24; C-statistic 0.71; Hosmer–Lemeshow *p* = 0.05. OR, odds ratio; CI, confidence interval. OR—odds ratio; CI—confidence interval.

**Table 8:** Adjusted odds ratios for 90-day mortality associated with treatment.

Treatment (Population)	*n/*Deaths	Crude OR (95% CI)	*p*	Adjusted OR (95% CI)	*p*
Oseltamivir (influenza)	1059/141	1.76 (1.11–2.80)	0.02	0.74 (0.44–1.24)	0.25
COVID-19 antivirals (COVID-19)	908/200	1.04 (0.76–1.42)	0.80	0.88 (0.63–1.22)	0.45
Any antibiotic (all mono-infected)	2118/379	3.85 (3.00–4.94)	<0.001	3.38 (2.58–4.43)	<0.001
Any antibiotic (COVID-19)	871/197	4.47 (3.17–6.30)	<0.001	4.03 (2.80–5.79)	<0.001
Any antibiotic (influenza)	1059/141	4.77 (3.10–7.36)	<0.001	3.33 (2.10–5.26)	<0.001
Any antibiotic (RSV)	188/41	1.38 (0.64–2.97)	0.42	1.19 (0.52–2.72)	0.67

Logistic regression for 90-day mortality among mono-infected patients. Adjusted models include age, sex, immunocompromised status and Charlson score (and virus type for the all-patient antibiotic model). Observational analysis without treatment-timing or severity-at-initiation data; associations are not causal. OR, odds ratio; CI, confidence interval.

## Data Availability

Data is available from the corresponding author with some restrictions to preserve anonymity of the subjects.

## Supplementary Materials

The following supporting information can be downloaded at: https://www.mdpi.com/article/10.3390/jcm15218483/s1, Figure S1: Patient flow diagram; Table S1: Sensitivity analyses: adjusted odds ratios (95% CI) for virus type with alternative adjustment sets (mono-infected patients unless stated); Table S2: Age-stratified analyses: adjusted odds ratios (95% CI) for virus type (mono-infected patients); Table S3: STROBE checklist for cohort studies.
