# Supplementary material for: Dynamics of Hospitalization and Outcomes of Patients with COVID-19, Influenza, and Respiratory Syncytial Virus (RSV) in a Large Hospital
Source: J Clin Med. 2026 Oct 31;15(21):8483. doi: 10.3390/jcm15218483 (PMC13764642; doi:10.3390/jcm15218483)
Supplement: Supplementary file 1 [file jcm-15-08483-s001.zip › jcm-4606997-supplementary.pdf]

Supplementary Material

Dynamics of Hospitalization and Outcomes of Patients with COVID-19, Influenza, and Respiratory Syncytial Virus (RSV) in a Large Hospital

Supplementary Figure S1. Patient flow diagram.

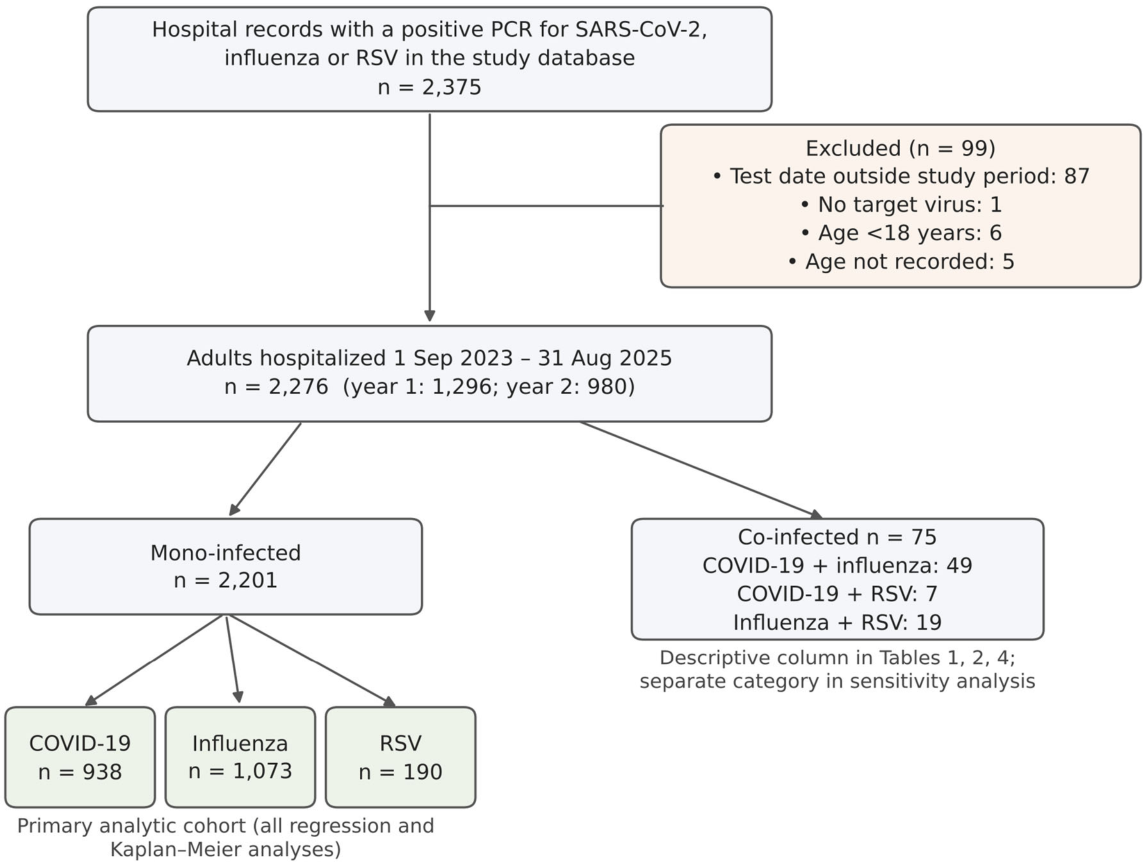

Records were excluded if the positive test date fell outside 1 September 2023 – 31 August 2025 (64 before, 23 after), if no target virus was detected, or if the patient was younger than 18 years or age was not recorded. Mono-infected patients form the primary analytic cohort; co-infected patients are shown separately in the descriptive tables and as a separate category in a sensitivity analysis.

Supplementary Table S1. Sensitivity analyses: adjusted odds ratios (95% CI) for virus type with alternative adjustment sets (mono-infected patients unless stated).

Outcome / model	n / events	COVID-19 vs. influenza	RSV vs. influenza	Co-infected vs. influenza	Overall p (virus)	C-statistic	HL p
**30-day mortality**							
Primary model	2,170 / 307	1.13 (0.86–1.49) p=0.37	1.26 (0.81–1.95) p=0.31	–	0.50	0.74	0.99
+ debilitation, home O2, prior hospitalization	2,166 / 307	0.99 (0.75–1.32) p=0.96	1.15 (0.73–1.80) p=0.56	–	0.81	0.79	0.02
+ study year	2,170 / 307	1.20 (0.90–1.60) p=0.21	1.29 (0.83–2.01) p=0.25	–	0.34	0.74	0.61
Co-infected as separate category	2,245 / 319	1.13 (0.86–1.49) p=0.38	1.27 (0.82–1.97) p=0.29	1.66 (0.84–3.30)	0.40	0.74	0.95
**90-day mortality**							
Primary model	2,170 / 388	1.28 (0.99–1.64) p=0.06	1.17 (0.77–1.77) p=0.46	–	0.16	0.74	0.92
+ debilitation, home O2, prior hospitalization	2,166 / 388	1.12 (0.86–1.45) p=0.40	1.07 (0.70–1.64) p=0.76	–	0.70	0.79	0.00
+ study year	2,170 / 388	1.39 (1.07–1.81) p=0.01	1.22 (0.80–1.85) p=0.35	–	0.05	0.75	0.41
Co-infected as separate category	2,245 / 400	1.28 (0.99–1.64) p=0.06	1.18 (0.78–1.79) p=0.43	1.29 (0.65–2.55)	0.29	0.74	0.91
**Composite endpoint**							
Primary model	2,008 / 368	0.87 (0.68–1.12) p=0.29	1.21 (0.80–1.84) p=0.37	–	0.24	0.71	0.05
+ debilitation, home O2, prior hospitalization	2,004 / 368	0.77 (0.59–1.01) p=0.06	1.10 (0.71–1.69) p=0.67	–	0.09	0.76	0.03
+ study year	2,008 / 368	0.89 (0.68–1.16) p=0.38	1.22 (0.80–1.86) p=0.35	–	0.30	0.71	0.19
Co-infected as separate category	2,081 / 381	0.87 (0.67–1.12) p=0.27	1.22 (0.81–1.86) p=0.34	1.21 (0.63–2.34)	0.31	0.71	0.15

All models include age, sex, immunocompromised status and Charlson score; reference: influenza. HL, Hosmer–Lemeshow goodness-of-fit test. The models adding debilitation, home oxygen and prior hospitalization showed lower HL p-values (HL $p<0.05$), indicating some miscalibration, although discrimination improved.

Supplementary Table S2. Age-stratified analyses: adjusted odds ratios (95% CI) for virus type (mono-infected patients).

Outcome / age group	n / events	COVID-19 vs. influenza	RSV vs. influenza	Overall p (virus)
**30-day mortality**				
<65 years	639 / 29	1.63 (0.69–3.84) p=0.27	2.92 (0.88–9.62) p=0.08	0.19
≥65 years	1,531 / 278	1.08 (0.81–1.44) p=0.60	1.08 (0.67–1.73) p=0.76	0.86
**90-day mortality**				
<65 years	639 / 37	1.78 (0.82–3.86) p=0.14	2.82 (0.93–8.58) p=0.07	0.13

Outcome / age group	n / events	COVID-19 vs. influenza	RSV vs. influenza	Overall p (virus)
≥65 years	1,531 / 351	1.22 (0.93–1.59) p=0.14	1.00 (0.64–1.57) p=0.99	0.30
**Composite endpoint**				
<65 years	555 / 45	0.83 (0.41–1.70) p=0.62	1.87 (0.64–5.46) p=0.25	0.37
≥65 years	1,453 / 323	0.88 (0.67–1.15) p=0.34	1.08 (0.69–1.71) p=0.73	0.50

Adjusted for age, sex, immunocompromised status and Charlson score; reference: influenza.

Supplementary Table S3. STROBE checklist for cohort studies.

Section / item	No.	STROBE recommendation (cohort studies)	Where reported in the revised manuscript
**Title and abstract**			
Title and abstract	1	(a) Indicate the study design with a commonly used term in the title or abstract; (b) provide an informative and balanced summary of what was done and what was found	Abstract (Methods: "retrospective cohort study"); Abstract Results and Conclusions
**Introduction**			
Background/rationale	2	Explain the scientific background and rationale for the investigation	Introduction, paragraphs 1–3
Objectives	3	State specific objectives, including any prespecified hypotheses	Introduction, final paragraph
**Methods**			
Study design	4	Present key elements of study design early in the paper	Methods – Study Design and Setting
Setting	5	Describe the setting, locations, and relevant dates, including periods of recruitment, exposure, follow-up, and data collection	Methods – Study Design and Setting (1 Sep 2023 – 31 Aug 2025); Outcome Definitions (30- and 90-day follow-up)
Participants	6	(a) Give the eligibility criteria, and the sources and methods of selection of participants; describe methods of follow-up	Methods – Study Population (inclusion/exclusion, co-infection classification); PCR Testing; Outcome Definitions
Variables	7	Clearly define all outcomes, exposures, predictors, potential confounders, and effect modifiers; give diagnostic criteria, if applicable	Methods – Data Collection; Outcome Definitions; Definitions (pneumonia, sepsis, septic shock – Sepsis-3)
Data sources/measurement	8	For each variable of interest, give sources of data and details of methods of assessment	Methods – PCR Testing; Data Collection (manual review of medical records); mortality was taken from the national population registry
Bias	9	Describe any efforts to address potential sources of bias	Methods – Statistical Analysis (restriction to mono-infected patients, a-priori covariates, sensitivity analyses); Discussion – limitations (testing criteria, documentation, residual confounding)
Study size	10	Explain how the study size was arrived at	Methods – Study Population (all eligible patients in the study period); Supplementary Figure S1; Discussion (power for the RSV comparison)
Quantitative variables	11	Explain how quantitative variables were handled in the analyses	Methods – Statistical Analysis (age and Charlson score as continuous terms; Box–Tidwell linearity check; age strata <65/≥65 years)

Section / item	No.	STROBE recommendation (cohort studies)	Where reported in the revised manuscript
Statistical methods	12	(a) Describe all statistical methods, including those used to control for confounding	Methods – Statistical Analysis
		(b) Describe any methods used to examine subgroups and interactions	Methods – Statistical Analysis (age-stratified analyses)
		(c) Explain how missing data were addressed	Methods – Statistical Analysis (complete-case analysis); table footnotes
		(d) If applicable, explain how loss to follow-up was addressed	Methods – Outcome Definitions [mortality ascertainment – confirm source, e.g. national population registry]
		(e) Describe any sensitivity analyses	Methods – Statistical Analysis (alternative adjustment sets, study year, co-infected category); Supplementary Table S1
**Results**			
Participants	13	(a) Report numbers of individuals at each stage of study; (b) give reasons for non-participation at each stage; (c) consider use of a flow diagram	Results – Patient Population; Supplementary Figure S1
Descriptive data	14	(a) Give characteristics of study participants; (b) indicate number of participants with missing data for each variable; (c) summarise follow-up time	Table 1 and footnote (missing data); Results – Patient Characteristics; follow-up 90 days (Methods)
Outcome data	15	Report numbers of outcome events or summary measures over time	Table 1 (30-/90-day mortality, composite); Figure 2 (Kaplan–Meier); Tables 3, 5–7 (events)
Main results	16	(a) Give unadjusted estimates and, if applicable, confounder-adjusted estimates and their precision (e.g., 95% CI); make clear which confounders were adjusted for; (b) report category boundaries when continuous variables were categorized; (c) consider translating relative risk into absolute risk	Tables 3, 5–8; Results – Predictors of Mortality; age strata defined (<65/≥65); absolute risks in Table 1 and Figure 2
Other analyses	17	Report other analyses done – e.g., analyses of subgroups and interactions, and sensitivity analyses	Results – sensitivity and age-stratified analyses; Supplementary Tables S1–S2
**Discussion**			
Key results	18	Summarise key results with reference to study objectives	Discussion, first paragraph
Limitations	19	Discuss limitations, taking into account sources of potential bias or imprecision; discuss both direction and magnitude of any potential bias	Discussion – limitations paragraph; precision of RSV estimates
Interpretation	20	Give a cautious overall interpretation considering objectives, limitations, multiplicity of analyses, results from similar studies, and other relevant evidence	Discussion; Conclusions
Generalisability	21	Discuss the generalisability (external validity) of the study results	Discussion – limitations (single-center design); future perspectives
**Other information**			

Section / item	No.	STROBE recommendation (cohort studies)	Where reported in the revised manuscript
Funding	22	Give the source of funding and the role of the funders for the present study	Funding statement reported in the acknowledgement

Checklist based on the STROBE Statement for cohort studies[13]. Locations refer to sections, tables and figures of the revised manuscript.
